# Patient satisfaction and quality of life with hemifacial spasm treatments in Finland's largest hospital district

**DOI:** 10.1016/j.bas.2025.105875

**Published:** 2025-11-17

**Authors:** Paula Palomäki, Johan Marjamaa, Tiina Sairanen, Mika Niemelä

**Affiliations:** a Hemifacial Spasm Research Group, Meilahti Tower Hospital, HUS Neurocenter, Haartmaninkatu 4, Helsinki, 00029, Finland; b Department of Neurosurgery, Meilahti Bridge Hospital, HUS Neurocenter, Haartmaninkatu 4, Helsinki, 00029, Finland; c Department of Neurology, Meilahti Tower Hospital, HUS Neurocenter, Haartmaninkatu 4, Helsinki, 00029, Finland

**Keywords:** Hemifacial spasm, Patient satisfaction, Quality of life, Cross-sectional studies, Surveys and questionnaires

## Abstract

**Introduction:**

Hemifacial spasm (HFS) treatments aim to remove involuntary facial twitching and improve the Quality of Life (QoL). No previous publications about overall patient treatment satisfaction or the impact of radiofrequency thermocoagulation (RFT) on patient satisfaction or QoL are available.

**Research question:**

This study describes patient satisfaction and QoL overall and in different treatment allocations.

**Material and methods:**

This cross-sectional questionnaire-based study was conducted in the Hospital District of Helsinki and Uusimaa (HUS). Consecutive HFS patients who had botulinum toxin (BTX), RFT, or microvascular decompression (MVD) between 2014 and 2019 were included. The participants answered HFS-7, 15D, and questions about treatment satisfaction and background.

**Results:**

59.40 % (N = 139) of contacted HFS patients in HUS (population responsibility 1.6 million) participated (82 female, 57 male). 79.14 % of patients were satisfied or very satisfied with the overall treatment. Most often, patients were satisfied with BTX (76.81 %). 52.63 % were satisfied with RFT, and 24.00 % with MVD. The mean HFS-7 scores for overall treatment, BTX, MVD, and RFT were 1.10, 1.13, 0.40, and 0.79 (scale 0–4). The mean 15D scores for overall treatment, BTX, MVD, and RFT were 0.87, 0.78, 0.91, and 0.86 (scale 0–1).

Having RFT correlated negatively to BTX treatment satisfaction (p < 0.001, effect size 0.449). Patients were more satisfied with RFT results if the duration of relief in facial twitching lasted longer (p0.034, effect size 0.580).

**Discussion and conclusion:**

Patients are satisfied with the overall treatment. Treatment satisfaction does not correlate with QoL. QoL after RFT seems comparable with other treatment allocations.

## Highlights

• Hemifacial spasm patients were satisfied with the overall treatment.
• Treatment satisfaction does not correlate with QoL.
• QoL after radiofrequency thermocoagulation is comparable with other treatments.

## Abbreviations:

HFS: Hemifacial spasm
BTX: Botulinum toxin
RFT: Radiofrequency thermocoagulation
MVD: Microvascular decompression
QoL: Quality of Life
HUS: The Hospital District of Helsinki and Uusimaa
HRQoL: Health-Related Quality of Life
SD: Standard deviation
CI: 95 % confidence interval

## 1 Introduction

Hemifacial spasm (HFS) is a movement disorder of muscles innervated by the facial nerve. It manifests as paroxysmal, synchronous, involuntary tonic, and clonic contractions of facial muscles (Lu et al., 2014). HFS is a rare neurovascular compression syndrome and facial movement disorder. The mean annual age-standardized incidence of HFS is 1.53, and the mean age-standardized yearly prevalence is 10.62 per 100,000 people (Nurminen et al., 2023). The treatment options, oral medications, botulinum toxin injections (BTX), radiofrequency thermocoagulation (RFT), and microvascular decompression (MVD) of HFS, aim to reduce or altogether remove the involuntary facial muscle contractions and improve the Quality of Life (QoL) of the HFS patient.

A handful of studies have been published about the patient satisfaction of HFS patients after BTX injections (Schnider et al., 1999; Cannon et al., 2010; Hsiung et al., 2002; Lasalvia et al., 2006; Lolekha et al., 2017; Birner et al., 1999). One study reports patient satisfaction after MVD (Illingworth et al., 1996). The QoL of HFS patients has been studied more than patient satisfaction. Previous publications cover the overall QoL with HFS and demonstrate the positive impact of BTX and MVD on HFS patients' QoL (Schnider et al., 1999; Tan, 2005; Wabbels and Yaqubi, 2021; Yuksel et al., 2019; Alciato et al., 2021; Heuser et al., 2007). However, there are no previous publications about overall patient treatment satisfaction and no publications about the impact of RFT on patient satisfaction or QoL.

This cross-sectional questionnaire-based study describes the overall patient satisfaction and QoL of HFS patients. It also aims to examine patient satisfaction and QoL separately for different treatment allocations, including BTX injections, MVD, and RFT.

## 2 Materials and methods

### 2.1 Standard protocol approvals, registrations, and patient consents

Approval for this study was obtained from the Hospital District of Helsinki and Uusimaa (HUS) Ethical Committee in July 2021. Participants gave informed written consent to participate in this study between March 7, 2021 and September 8, 2022.

### 2.2 Patient cohort

All consecutive patients with HFS diagnosis (ICD-10: G51.31, G51.32, or G51.33) and who had BTX injections, RFT, or MVD between 2014 and 2019 were identified from the electronic medical records of the HUS (population responsibility of 1.6 million residents). A neurologist, otolaryngologist, or neurosurgeon has confirmed all HFS diagnoses. Patients who had passed away or did not have contact information in the electronic medical records system were excluded from the study. The remaining patients were contacted. First, each patient received a research participation package, including an information letter to participants, questionnaires, and a patient consent form. If the patient did not respond to the research invitation, the same research participation package was sent again after a few months. After that, the patient received a text message reminder to respond to the research invitation and to follow three calls from the research staff, with the option to participate via call interview or refuse to participate.

### 2.3 Data collection

A STROBE checklist for cross-sectional studies was used to ensure that this article meets the EQUATOR Network Reporting Guidelines. Clinical background information and treatment paths from early symptoms and diagnosis up to 2020 were collected for each HFS patient from electronic patient records regarding sex, side of the spasm, age of diagnosis, and HFS treatment allocations. Participation in this cross-sectional questionnaire-based study included answering two questionnaires, HFS-7 and 15D, and some unvalidated questions selected by the research group, and returning them back to the research group with written consent to participate in this study.

HFS-7 is a disease-specific short (7-dimensional) self-rating outcome instrument for measuring QoL with HFS using a scale from 0 to 4. (Tan, 2005). A higher score indicates a lower QoL. 15D is a generic comprehensive (15-dimensional) self-administered outcome instrument for measuring Health-Related Quality of Life (HRQoL) among adults (age 16+) (Sintonen, 2001). A higher single index score indicates a higher HRQoL. The selection of additional questions about treatment history, treatment satisfaction with HFS, and background was made based on the Health 2011 survey. The Health 2011 survey was coordinated by the Finnish Institute for Health and Welfare (THL) in cooperation with an extensive network of specialists. It aimed to produce reliable data on the health, functional capacity, and welfare of the Finnish population and its subgroups and any changes in these. Treatment satisfaction was assessed in terms of overall satisfaction and by the type of treatment.

### 2.4 Statistical analysis

The statistical analysis was performed using IBM SPSS Statistics v28.0 and Microsoft Excel v2018 software. Counts (N) and percentages (%) were used to describe the categorical variables. Means, standard deviations (SD), and 95 % confidence intervals (CI) were calculated to define continuous variables.

When analyzing QoL, patients were divided into different treatment categories based on the latest treatment type. The data collected from the 15D questionnaire was first cleaned by filling in missing values using multivariate imputation utilizing a SPSS algorithm developed by the 15D developers. In 15D responses, five had one missing item, and one had two. Then, the data was interpreted using an algorithm developed for the questionnaire. The algorithm uses a set of utility or preference weights to generate the 15D score, a single index number on a scale of 0–1. For HFS-7, no official imputation protocol has been published. In three HFS-7 responses, one item was missing. When a participant was missing one item, the HFS-7 score was calculated as the mean of the available six items (person-mean imputation) (Mcdonald et al., 2000).

Four variables were converted to dichotomous nominal variables. Daily and sporadic smoking was considered as current smoking. The answers to the questions about current alcohol consumption were converted to a dichotomous nominal variable according to Finnish care guidelines updated in 2018. The categories were defined as moderate to high alcohol risk consumption and low alcohol risk consumption. The patients were asked about treatment satisfaction using a five-level bipolar Likert-type ordinal scale. The dichotomous nominal treatment satisfaction categories were defined as one to two (very satisfied to somewhat satisfied) and three to five (not sure to extremely unsatisfied). During data analysis, the questionnaire's original patient satisfaction rating scale was inverted to run from five (very satisfied) to 1 (very unsatisfied) to present the results more logically. In the original questionnaire, one represented very satisfied, and five indicated very unsatisfied. Education level was recorded using an eleven-step ordinal scale. The answers were divided into high and low education levels. A bachelor's, Master's, or Doctoral degree was considered a high education level, and other reported lower education levels were regarded as low.

In inferential statistics, p < 0.05 was used as a threshold for statistically significant differences between groups. The statistical significance between two dichotomous variables was analyzed using Fisher's exact test and effect size with the Phi coefficient. Comparisons between continuous variables and dichotomous nominal variables were first prepared by testing the distribution normality of the continuous variable with the Shapiro-Wilk test and equality of variances based on means with Levene's test. Statistical differences between the two groups were tested with two-sample t-tests or the Mann-Whitney *U* test. Cohen's d or Eta squared was used for the effect size. Pearson correlation and Pearson correlation coefficient were used to test the linear relationships between continuous variables. Given the exploratory nature of these comparisons and the relatively small subgroup sizes, no formal correction for multiple comparisons (e.g., Bonferroni adjustment) was applied. In Table 3, Table 4, Table 5, Table 6, Table 7, statistically significant p-values are highlighted with an asterisk (∗). Only statistically significant comparisons include corresponding effect sizes, which are interpreted qualitatively as trivial, weak, moderate, or strong to aid clinical interpretation.

### 2.5 Data availability

The anonymized data supporting this study's findings are available on request from the corresponding author, P. Palomäki. The data are not publicly available because they include information that could compromise the privacy of research participants.

## 3 Results

**Fig. 1:**
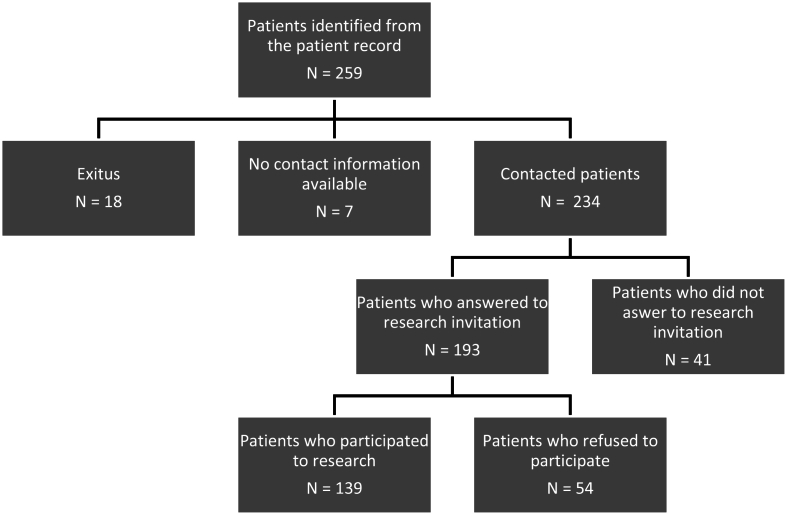
Flowchart of patient identification, exclusion, and inclusion in the study.

The flow of patient identification, exclusion, and inclusion is summarized in Fig. 1. 59.40 % of contacted HFS patients in the HUS population responsibility area participated in this study by completing the questionnaires and a written consent form (N = 139, 82 female and 57 male). Table 1 shows the patient cohort's clinical characteristics and self-reported treatment satisfaction. Patients were surveyed with a mean of 11 years after the diagnosis of HFS. Women outnumbered men among participants (58.99 %). The most common self-reported comorbidities were hypertension and musculoskeletal conditions. All but one patient had had botulinum toxin injections, 25 had undergone MVD, and 19 had had RFT. The overall mean patient satisfaction score on a scale of 1–5 was 4.04 (0.95 SD). The number of patients satisfied (very satisfied to somewhat satisfied) varied between different treatment allocations. The mean patient satisfaction with BTX was 3.88 (1.11 SD), MVD 2.29 (1.60 SD), and RFT 3.50 (1.58 SD).

**Table 1:** Clinical characteristics and self-reported treatment satisfaction for hemifacial spasm and its different treatment allocations (N = 139).

Nominal variable	N	%
Female	82	58.99
Low education level∗^1^	85	61.15
Current smoking∗^2^	20	14.39
Moderate to high alcohol risk consumption∗^3^	69	49.64
BTX treatment tested	138	99.28
- BTX treatment pauses lasting over six months∗^4^	59	41.73
MVD done∗^5^	25	17.99
- BTX treatment after MVD	13	52.00
RFT done∗^6^	19	13.67
- BTX treatment after RFT	5	26.32
Self-reported long-term medical conditions∗^7^		
- Hypertension	78	56.12
- Diabetes mellitus	20	14.39
- Cardiovascular disease	23	16.55
- Psychiatric condition	23	16.55
- Other neurological condition	25	17.99
- Musculoskeletal condition	47	33.81
- Cancer	11	7.91
- Sence organ disorder	22	15.83
Patient's treatment satisfaction (satisfied or very satisfied)		
- Overall hemifacial spasm treatment (139 patients) ∗^8^	110	79.14
- BTX injections (138 patients) ∗^9^	106	76.81
- MVD (25 patients) ∗^10^	6	24.00
- RFT (19 patients) ∗^11^	10	52.63
**Continuous variable**	**Mean**	**SD (CI)**

Age at the time of participating in this study (years)∗^12^	66.84	12.94 (64.66–69.03)
Time from the hemifacial spasm diagnosis (years)∗^13^	11.23	7.38 (9.99–12.48)
Time smoking daily (years)∗^14^	9.65	15.54 (7.00–12.31)
Time from the beginning of symptoms to 1st MVD (years)∗^15^	6.77	5.28 (4.54–9.00)
Number of BTX treatment pauses lasting over six months∗^16^	0.68	1.51 (0.40–0.96)
Number of RFT treatments∗^17^	0.47	1.81 (0.17–0.78)
Duration of RFT benefit (months)	9.79	8.47 (5.71–13.87)

datapoints missing: ∗^1^ 2 (1.44 %), ∗^2^ 1 (0.72 %), ∗^3^ 2 (1.44 %), ∗^4^ 6 (4.35 %), ∗^5^ 2 (1.44 %), ∗^6^ 2 (1.44 %), ∗^7^ 4 (2.88 %), ∗^8^ 3 (2.16 %), ∗^9^ 2 (1.45 %), ∗^10^ 1 (4.00 %), ∗^11^ 1 (5.26 %), ∗^12^ 2 (1.44 %), ∗^13^ 2 (1.44 %), ∗^14^ 5 (3.60 %), ∗^15^ 1 (4:00 %), ∗^16^ 24 (17.27 %), ∗^17^ 2 (1.44 %).

BTX: Botulinum toxin.

MVD: Microvascular decompression.

RFT: Radiofrequency thermocoagulation.

Table 2 presents the results of the disease-specific HFS-7 questionnaire and the health-related 15D questionnaire's QoL results. Overall QoL measured with HFS-7 (1.10) and 15D (0.87) was high. Patients who had had MVD had the highest disease-specific and health-related QoL.

**Table 2:** The results of HFS-7 (disease-specific QoL questionnaire, scale 0–4) and 15D (generic comprehensive HRQoL questionnaire, scale 0–1).

HFS-7	Mean (SD)	CI	Participants	Datapoints cleaned N (%)
Overall	1.10 (1.19)	1.03–1.18	139	3 (0.31)
BTX	1.13 (1.19)	1.04–1.21	120	3 (0.36)
MVD	0.40 (0.89)	0.25–0.78	5	0 (0.0)
RFT	0.79 (1.19)	−0.71–1.51	14	0 (0.0)

15D	Mean (SD)	CI	Participants	Datapoints cleaned N (%)
Overall	0.87 (0.10)	0.85–0.89	139	7 (0.34)
BTX	0.87 (0.11)	0.85–0.89	120	7 (0.39)
MVD	0.91 (0.04)	0.86–0.96	5	0 (0.0)
RFT	0.86 (0.06)	0.83–0.89	14	0 (0.0)

BTX: Botulinum toxin.

MVD: Microvascular decompression.

RFT: Radiofrequency thermocoagulation.

Treatment satisfaction rates and QoL scores did not follow the same pattern across treatments. As shown in Table 1, Table 2, patients after MVD reported the lowest satisfaction rate (24 %) despite the best disease-specific and generic QoL scores (HFS-7 = 0.40, 15D = 0.91). Patients treated with BTX injections showed the highest satisfaction (76.8 %) but lower QoL scores (HFS-7 = 1.13, 15D = 0.87). The radiofrequency thermocoagulation (RFT) group showed intermediate values for both satisfaction (52.6 %) and QoL (HFS-7 mean 0.79; 15D mean 0.86).

Table 3, Table 4 demonstrate the correlations between patient characteristics and treatment paths with treatment satisfaction overall and in different treatment allocations. In Table 3, low satisfaction with BTX treatment was associated with pauses in BTX injections over a six-month period. Commitment to BTX injections again after MVD or the patient had had RFT was also associated with low patient satisfaction with BTX treatment. In Table 4, patients who had lived longer with an HFS diagnosis tended to report higher satisfaction with the results of MVD (p = 0.043, Cohen's d 1.014, 1.97–0.033 CI). Longer relief from facial twitching was associated with higher patient satisfaction with RFT results (p = 0.034, Eta^2 0.580).

**Table 3:** Correlation of dichotomous patient or treatment characteristics and dichotomous self-reported patient satisfaction stratified by treatments.^a^

Characteristic	ALL-OVER		BTX		MVD		RFT	
	p-value	Effect size	p-value	Effect size	p-value	Effect size	p-value	Effect size
Sex	1.000	–	0.402	–	0.640	–	0.664	–
BTX pause over six months	0.006^b^	−0.251 (weak)	<0.001^b^	−0.354 (moderate)	0.292	–	0.206	–
MVD done	0.092	–	0.005^b^	−0.264 (weak)	–	–	0.314	–
BTX after MVD	0.411	–	0.038^b^	0.497 (strong)	0.155	–	0.592	–
RFT done	0.057	–	<0.001^b^	−0.449 (strong)	0.061	–	–	–
BTX after RFT	1.000	–	0.290	–	1.000	–	0.007	–
Education level^b^^1^	0.501	–	0.830	–	0.640	–	1.000	–
Cardiovascular disease^b^^2^	0.551	–	1.000	–	0.532	–	1.000	–
High blood pressure	0.105	–	1.000	–	0.655	–	0.633	–
Diabetes mellitus	0.752	–	0.558	–	1.000	–	0.118	–
Psychiatric condition	1.000	–	1.000	–	1.000	–	0.604	–
Neurological condition	0.235	–	0.097	–	1.000	–	0.118	–
Musculoskeletal condition^b^^3^	0.473	–	1.000	–	1.000	–	1.000	–
Cancer	0.100	–	0.235	–	0.481	–	1.000	–
Sense organ disorder	0.002^b^	−0.292 (weak)	0.157	–	0.133	–	0.518	–
Current smoking	0.532	–	1.000	–	0.539	–	0.537	–
Moderate to high alcohol risk consumption^b^^4^	0.266	–	0.526	–	0.155	–	1.000	–

∗^1^High education level = Bachelor's, Master's, or Doctoral degree; low education level = other reported lower education levels.

∗^2^For example, chronic arrhythmias, coronary heart disease, and stroke.

∗^3^For example, osteoporosis, arthrosis, rheumatic conditions, symptomatic back, and intervertebral disc degeneration.

∗^4^Levels of alcohol risk consumption have been defined based on Finnish care guidelines.

BTX: Botulinum toxin.

MVD: Microvascular decompression.

RFT: Radiofrequency thermocoagulation.

a Statistical tests used: Fisher's exact test (p-value), Phi coefficient (effect size, scale −1 to 1).

b Statistically significant test result.

**Table 4:** Comparison of continuous baseline characteristics and dichotomous self-reported treatment satisfaction.^a^

Characteristic	Normal distribution	Equality of variances	P-value	Effect size
Overall				

Age at the time of study	0.749	0.383	0.351	–
Years with hemifacial spasm diagnosis	0.144	0.727	0.498	–
Years of smoking daily	<0.001^b^	0.869	0.523	–
Number of BTX pauses over six months	0.017^b^	0.094	0.086	–
Number of RFT treatments	<0.001^b^	0.374	0.038^b^	0.004 trivial
Duration of RFT benefit (months)	0.370	0.125	0.187	–

Botulinum toxin				

Age at the time of study	0.749	0.969	0.598	–
Years with hemifacial spasm diagnosis	0.144	0.536	0.666	–
Years of smoking daily	<0.001^b^	0.501	0.460	–
Number of BTX pauses over six months	0.017^b^	0.010^b^	0.028^b^	0.048 trivial
Number of RFT treatments	<0.001^b^	<0.001^b^	<0.001^b^	0.150 trivial
Duration of RFT benefit (months)	0.370	0.084	0.307	–

Microvascular decompression				

Age at the time of study	0.749	0.173	0.262	–
Years with hemifacial spasm diagnosis	0.144	0.115	0.043^b^	1.014 strong
Years of smoking daily	<0.001^b^	0.150	0.431	–
Number of BTX pauses over six months	0.017^b^	0.597	0.295	–
Number of RFT treatments	<0.001^b^	0.096	0.090	–
Duration of RFT benefit (months)	0.370	–	–	–

Radiofrequency thermocoagulation				

Age at the time of study	0.749	0.323	0.938	–
Years with hemifacial spasm diagnosis	0.144	0.941	0.738	–
Years of smoking daily	<0.001^b^	0.033^b^	0.161	–
Number of BTX pauses over six months	0.017^b^	0.559	0.247	–
Number of RFT treatments	<0.001^b^	0.074	0.203	–
Duration of RFT benefit (months)	<0.001^b^	0.050	0.034^b^	0.580 strong

BTX: Botulinum toxin.

MVD: Microvascular decompression.

RFT: Radiofrequency thermocoagulation.

a Statistical test used: Shapiro-Wilk test (normal distribution of continuous variable), Levene's test (equality of variances based on means), Two-Sample *t*-test or Mann Whitney *U* test (statistical significance), Cohen's d or Eta^2^ (effect size).

b Statistically significant test result.

Table 5, Table 6, Table 7 show the correlation of self-reported disease-specific (HFS-7) and HRQoL (15D) with varying patient characteristics. Receiving BTX after RFT was strongly associated with lower disease-specific QoL (p = 0.015, Eta^2 0.259).

**Table 5:** Correlation of self-reported disease-specific quality of life (HFS-7) with dichotomous baseline characteristics.^a^

Characteristic and QoL	Normal distribution	Equality of variances	P-value	Effect size
Sex	<0.001^b^	0.905	0.962	–
BTX pause over six months	<0.001^b^	0.082	0.206	–
MVD done	<0.001^b^	0.029^b^	0.102	–
BTX after MVD	<0.001^b^	0.891	0.437	–
RFT done	<0.001^b^	0.012^b^	0.211	–
BTX after RFT	<0.001^b^	0.294	0.015^b^	0.259 strong
Education level	<0.001^b^	0.656	0.256	–
Cardiovascular disease	<0.001^b^	0.690	0.547	–
High blood pressure	<0.001^b^	0.187	0.615	–
Diabetes mellitus	<0.001^b^	0.085	0.292	–
Psychiatric condition	<0.001^b^	0.626	0.653	–
Neurological condition	<0.001^b^	0.706	0.010^b^	0.048 weak
Musculoskeletal condition	<0.001^b^	0.167	0.666	–
Cancer	<0.001^b^	0.993	0.518	–
Sense organ disorder	<0.001^b^	0.739	0.105	–
Current smoking	<0.001^b^	0.042^b^	0.092	–
Moderate to high alcohol risk consumption	<0.001^b^	0.105	0.513	–

BTX: Botulinum toxin.

MVD: Microvascular decompression.

RFT: Radiofrequency thermocoagulation.

a Statistical test used: Shapiro-Wilk test (normal distribution of continuous variable), Levene's test (equality of variances based on means), Two-Sample *t*-test or Mann Whitney U (statistical significance), and Cohen's d or Eta^2^ (effect size).

b Statistically significant test result.

**Table 6:** Correlation of self-reported health-related quality of life (15D) with dichotomous baseline characteristics.^a^

Characteristic and QoL	Normal distribution	Equality of variances	P-value	Effect size
Sex	<0.001^b^	0.166	0.395	–
BTX pause over six months	<0.001^b^	0.530	0.059	–
MVD done	<0.001^b^	0.051	0.055	–
BTX after MVD	<0.001^b^	0.020^b^	0.168	–
RFT done	<0.001^b^	0.041^b^	0.026^b^	0.012 trivial
BTX after RFT	<0.001^b^	0.363	0.081	–
Education level	<0.001^b^	0.872	0.090	–
Cardiovascular disease	<0.001^b^	0.593	0.038^b^	0.030 weak
High blood pressure	<0.001^b^	0.033^b^	0.002^b^	0.067 trivial
Diabetes mellitus	<0.001^b^	0.352	0.053	–
Psychiatric condition	<0.001^b^	0.012^b^	<0.001^b^	0.138 trivial
Neurological condition	<0.001^b^	0.002^b^	0.037^b^	0.059 trivial
Musculoskeletal condition	<0.001^b^	0.833	0.025^b^	0.025 weak
Cancer	<0.001^b^	0.937	0.228	–
Sense organ disorder	<0.001^b^	0.279	<0.001^b^	0.109 moderate
Current smoking	<0.001^b^	0.800	0.889	–
Moderate to high alcohol risk consumption	<0.001^b^	0.357	0.415	–

BTX: Botulinum toxin.

MVD: Microvascular decompression.

RFT: Radiofrequency thermocoagulation.

a Statistical test used: Shapiro-Wilk test (normal distribution of continuous variable), Levene's test (equality of variances based on means), Two-Sample *t*-test or Mann Whitney U (statistical significance), and Cohen's d or Eta^2^ (effect size).

b Statistically significant test result.

**Table 7:** Correlation of continuous patient or treatment characteristics with self-reported disease-specific (HFS-7) and health-related quality of life (15D) results.^a^

Variable	HFS-7		15D	
	P-value	Effect size	P-value	Effect size
Age at the time of study	0.071	−0.155	0.060	−0.161
Years with hemifacial spasm diagnosis	0.151	0.123	0.231	−0.103
Years of smoking daily	0.626	−0.043	0.265	−0.097
Number of BTX pauses over six months	0.553	−0.056	0.769	0.028
Number of RFT treatments	0.005^b^	0.241 weak	0.697	−0.034
Duration of RFT benefit (months)	0.072	−0.422	0.097	0.391

BTX: Botulinum toxin.

RFT: Radiofrequency thermocoagulation.

a Statistical test used: Pearson correlation (p-value) and Pearson correlation coefficient (effect size).

b Statistically significant test result.

## 4 Discussion

This study reports patient satisfaction and QoL of HFS patients after RFT that are comparable with the results of other HFS treatment allocations. Compared to other patient satisfaction studies done for HFS patients, this is on the larger side of studies investigating HFS patient satisfaction with 139 participating patients (Lee et al., 2022). 59.40 % of contacted HFS patients in the HUS population responsibility area (1.6 million residents) answered the questionnaires of this study, indicating a high participation rate. The patient population in this study is a subpopulation of our more extensive epidemiological study on the epidemiology of HFS. The sex distribution of this study is very similar to our previous study in which 55.52 % of the mean age-standardized yearly prevalence of HFS were women (Nurminen et al., 2023). In this study, 58.99 % of patients were female. This study made it possible to compare RFT with other HFS treatment options for the first time. It also revealed HFS patients' treatment satisfaction and QoL in the Finnish health care system, where treatment visits are heavily supported by the government and, therefore, lower the threshold to adhere to treatment plans suggested by the professionals.

In this study, 79.14 % of patients were satisfied or very satisfied with the overall treatment. 52.63 % of patients were satisfied with the results of RFT. Previous research found that patients’ satisfaction with RFT treatment is connected to the duration of relief from facial twitching (Palomäki et al., 2024). In this study, the duration of RFT benefit was also the best positive indicator of patient satisfaction after RFT. The most satisfied patients were with the results of BTX injections (76.81 %). Previous publications have mixed results but seem to support this finding overall. Two previous publications reported 90–100 % patient satisfaction with BTX injections (Schnider et al., 1999; Cannon et al., 2010). Three articles on patient satisfaction with BTX injections had similar results to this study (Hsiung et al., 2002; Lasalvia et al., 2006; Lolekha et al., 2017). One study reported much lower patient satisfaction with BTX injections (50 %) than this study (Birner et al., 1999). In this study, only 24.00 % of patients were satisfied with the results of MVD and more years spent with hemifacial spasm diagnosis associated positively with patient satisfaction after MVD (p 0.043, effect size 1.014). The only previous publication related to patient satisfaction after MVD reports a much higher 91 % patient satisfaction in a larger study population of 83 patients with a similar study method of cross-sectional retrospective survey sent all HFS patients in regional cohort, similar operative technique (posterior fossa approach as described by Jannetta and modified by Sugita), similar mean follow-up period of eight years, and symptom duration of a mean of 6.15 years (this study 6.77 years) before surgery (Illingworth et al., 1996). Some notable underlying factors should be considered when interpreting the discrepancy in patient satisfaction after MVD between these two studies. First, Illighworth et al. did not address the exact type of survey sent to the patients. The article provides only dichotomous results (satisfied or unsatisfied). In this paper, patient satisfaction was initially assessed using a 5-level bipolar ordinal Likert-type scale, which was converted to a scale ranging from "satisfied" (1–2, extremely satisfied to somewhat satisfied) to "unsatisfied" (3–5, not sure to extremely unsatisfied). Second, patient expectations may differ. Patients may consider MVD as “the last resort” and be disappointed if it does not lead to complete symptom resolution. Thirdly, a higher complication rate or the occurrence of permanent complications may reduce treatment satisfaction, even if the spasm resolves. Lastly, in some cases, complete decompression is not possible due to anatomical constraints. For example, the compressing vessel may lie between the facial nerve and the vestibulocochlear nerve, making the complete and certain decompression impossible.

The overall HFS-7 score for all patients was a mean of 1.10 and a median of 1. In 2005, Tan et al. reported that their study population had an HFS-7 median of 2, slightly higher and indicating lower disease-specific QoL. (Tan, 2005). In contrast, this study's mean HFS-7 score for patients with BTX injections was 1.13. 2019 Yuksel et al. reported a mean HFS-7 score of 0.77 (31.86 % lower) four weeks after BTX treatment (Yuksel et al., 2019). Many studies highlight the significant positive impact of BTX injections on the QoL (Schnider et al., 1999; Wabbels and Yaqubi, 2021; Kongsaengdao et al., 2021; Rudzińska et al., 2012; Setthawatcharawanich et al., 2011; Reimer et al., 2005). Even though patients were not satisfied with treatment results after MVD in this study, they had the best disease-specific QoL (lowest HFS-7 score) compared to other treatment allocations, with a mean of 0.40 or median 1 in this study. Multiple other studies postoperatively have had slightly better results (a median of 0–0.5 or a mean of 0.1) (Alciato et al., 2021; Heuser et al., 2007; Compagnon et al., 2021; Teton et al., 2020). Some studies reported slightly higher scores postoperatively (mean 2 to 2.2), indicating lower QoL (Montava et al., 2016; Lawrence et al., 2018; Ray et al., 2010). Like BTX treatment, MVD has proven to increase QoL in patients suffering from HFS in multiple studies (Alciato et al., 2021; Heuser et al., 2007; Teton et al., 2020; Montava et al., 2016; Lawrence et al., 2018; Trashin et al., 2023). Yi et al. found that education level correlated statistically significantly with the general health, social function, and role-emotional domains of SF-36 (Yi et al., 2022). The current study found no relevant correlation between education level and HFS-7 or 15D scores.

The observed disconnect between treatment satisfaction and quality of life highlights the complexity of patient-reported outcomes in HFS. Despite the lowest satisfaction levels, MVD patients exhibited the best QoL scores, while BTX patients were most satisfied but had lower QoL. Patients undergoing MVD might have high objective benefit but lingering dissatisfaction if recovery was prolonged or expectations of a complete cure were unmet. Conversely, BTX injections, being minimally invasive and easily accessible in Finland's public healthcare system, may generate higher satisfaction even when overall QoL remains modest. These findings reinforce the idea that satisfaction and QoL represent complementary but distinct dimensions of patient-centered outcomes.

Although statistically significant associations were observed in this study, some findings should be interpreted with caution due to limited statistical power. For example, the positive correlation between longer duration of HFS diagnosis and higher satisfaction among MVD patients (p = 0.043, Cohen's d = 1.014) appears large in magnitude, but the small number of MVD patients (N = 25) limits the precision of this estimate and increases the risk of both type I and type II errors. Similarly, patient satisfaction with RFT was higher when relief from facial twitching lasted longer. Although this trend is consistent with prior literature, the small sample size of RFT patients (N = 19) reduces the robustness of this finding. Other associations, such as lower disease-specific QoL among patients receiving BTX after RFT, also demonstrated statistically significant effect sizes but should be interpreted cautiously due to small subgroup sizes and potential confounding factors. Across these analyses, small numbers of participants in certain treatment categories, combined with the cross-sectional design, reduce the certainty of statistical associations and make the reported differences more susceptible to sampling variability. Therefore, while the observed associations provide valuable preliminary insights, they should be confirmed in larger, prospective studies. In addition, because multiple subgroup comparisons were performed, we considered but did not apply formal corrections for multiple testing, such as the Bonferroni adjustment. Applying strict correction procedures in this context could markedly inflate the risk of type II errors, potentially obscuring meaningful associations in small subgroups. Therefore, p-values are presented as descriptive indicators of statistical trends rather than confirmatory evidence, and all findings should be interpreted cautiously.

As a questionnaire-based cross-sectional study, there is an inevitable selection bias of patients as, for example, busy patients, patients with highly disabling conditions, or patients with very low overall patient satisfaction or QoL may not participate in this type of study. Recall bias may have influenced patient responses, as the survey was administered a mean of 11 years after initial diagnosis. Nevertheless, the long follow-up period, individual treatment paths, and likely multiple treatment sessions over the years also provide valuable insight into real-world treatment impressions. Most patients have also self-administered the questionnaires with no low threshold possibility to ask for guidance. It is also important to highlight that the Finnish and Swedish translations of HFS-7 have not undergone formal validation. Although we performed professional forward–backward translation of HFS-7 in both languages, the lack of formal validation may affect the reliability and comparability of the disease-specific QoL scores. Additionally, the satisfaction questions were specifically designed for this study and were not based on standardized or previously validated instruments. Therefore, our results regarding disease-specific QoL and patient satisfaction should be interpreted with caution until validated and standardized Finnish/Swedish tools are available. Missing data in the 15D questionnaire were imputed using an algorithm provided by the 15D developers to preserve sample size and maintain comparability between groups. Missing data in the HFS-7 questionnaire were imputed using person-mean imputation. These procedures may have reduced variability and slightly affected the accuracy of QoL estimates.

## 5 Conclusions

Overall, HFS patients are satisfied with the treatment. Treatment satisfaction for different treatment allocations varies, and it does not seem to be connected to QoL. Patients are pretty content with the results of RFT. In this cohort, QoL after RFT was comparable to QoL after BTX injections, supporting its role as a treatment option for HFS; however, prospective studies are needed to confirm these findings.

## Declaration of generative AI and AI-assisted technologies in the manuscript preparation process

During the preparation of this work the author(s) used Grammarly Pro AI Writing Assistance in order to aid with grammar and improve readability, but not to generate original text. After using this service, the author(s) reviewed and edited the content as needed and take(s) full responsibility for the content of the published article.

## Declaration of competing interest

The authors declare the following financial interests/personal relationships which may be considered as potential competing interests: Paula Palomaki reports financial support was provided by the Department of Research and Development of Helsinki University Neurocenter. If there are other authors, they declare that they have no known competing financial interests or personal relationships that could have appeared to influence the work reported in this paper.
